# Guided implant surgery with modification of the technique involving the raising of a semicircular miniflap: A preliminary study

**DOI:** 10.4317/medoral.17363

**Published:** 2012-05-01

**Authors:** María Peñarrocha, José Viña, Laura Maestre, David Peñarrocha, José Balaguer

**Affiliations:** 1 Associate Professor of Oral Surgery. Professor of the Master in Oral Surgery and Implantology. Valencia University Medical and Dental School; 2 Degree in Dental Surgery. Master in Oral Surgery and Implantology. Valencia University Medical and Dental School; 3 Degree in Dental Surgery. Resident of the Master in Oral Surgery and Implantology. Valencia University Medical and Dental School. Valencia (Spain); 4 Associate Professor of Oral Surgery. Professor of the Master in Oral Surgery and Implantology. Valencia University Medical and Dental School

## Abstract

Objective: An evaluation is made of pain, swelling and peri-implant attached mucosal width after implant-based rehabilitation involving guided surgery and a modification of the technique with the raising of a semicircular miniflap, in single and partial replacements. Study design: A case-control study was carried out. The study group consisted of 12 patients with the placement of 19 implants using a guided surgery and miniflap technique. The control group consisted of 12 patients with the placement of 22 implants using the conventional technique. Each patient scored postoperative swelling and pain by means of a visual analog scale (VAS). Attached vestibular mucosa width was evaluated 12 weeks after implant placement. Results: Twelve operations were carried out in each group. Immediate aesthetics were established for all implants of the study group. One implant failed in each group. Maximum pain was recorded after 6 hours in both groups (mean VAS score 4 and 4.9 in the study and control group, respectively). Maximum swelling was recorded after 24 hours (mean VAS score 2.5) in the study group and on the second day (mean VAS score 3.4) in the control group. The mean attached vestibular mucosa width was 2.9 mm in the study group and 3.2 mm in the control group. Conclusion: In this preliminary study, guided implant surgery with a semicircular miniflap in single and partial replacements resulted in slightly less postoperative pain and swelling than with the conventional implant technique. The attached vestibular mucosa width was greater in the control group, though the differences were very small.

** Key words:**Guided surgery, flapless surgery, miniflap, peri-implant mucosa.

## Introduction

Dental implant placement through guided surgery offers the advantages of minimally invasive surgery, e.g., lesser postoperative morbidity and a shortening of the surgical times (1). Flapless surgery preserves the periosteum and blood supply to the bone (2,3), avoids modifications in gingival marginal contour (2,3), reduces bleeding (2,3), and increases the success of immediate loading thanks to maintenance of the blood supply (2). Flapless surgery is blind in the sense that it is difficult to evaluate the contours and angulations of the alveolar margin – the technique therefore being limited to cases with great bone width (4). This concept has changed in recent years thanks to implant placement under guided surgery (5). One of the inconveniences of guided flapless surgery is that soft tissue distribution is not possible (6).

A keratinized gum width of 2 mm around the teeth is recommended in order to preserve periodontal tissue health (7,8). Until only a few years ago there was controversy over the need for attached gingival tissue around implants to ensure peri-implant health (9). Recent studies in humans (9,10) and animals (11) have shown that an insufficient peri-implant keratinized mucosal width favors plaque accumulation and lingual bleeding (9), vestibular recessions (9-11), and crestal bone loss (10,11).

The present preliminary study was designed to evaluate pain, swelling and peri-implant attached mucosal width after implant-based rehabilitation involving guided surgery and a modification of the technique with the raising of a semicircular miniflap, in single and partial replacements.

## Material and methods

-Patient screening

A case-control study was carried out among patients requiring single or partially edentulous segment rehabilitation through implant-based guided surgery in the Oral Surgery Unit of a University Dental Clinic. The study group consisted of 12 patients (3 males and 9 females) subjected to 12 guided operations with a modification of the technique involving the raising of a semicircular miniflap. The mean age was 42 years (range 30-58). The control group in turn consisted of 12 patients (4 males and 8 females with a mean age of 38 years; range 26-53) subjected to implant rehabilitation with the conventional surgical technique.

**Figure 1:**
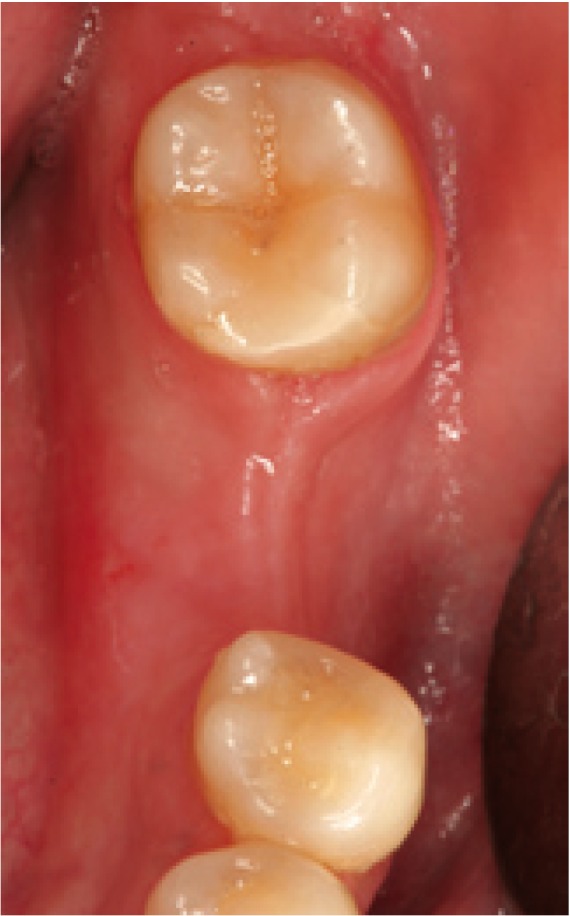
Preoperative view. Note the insufficient attached mucosa for performing surgery with the circular scalpel.

**Figure 2:**
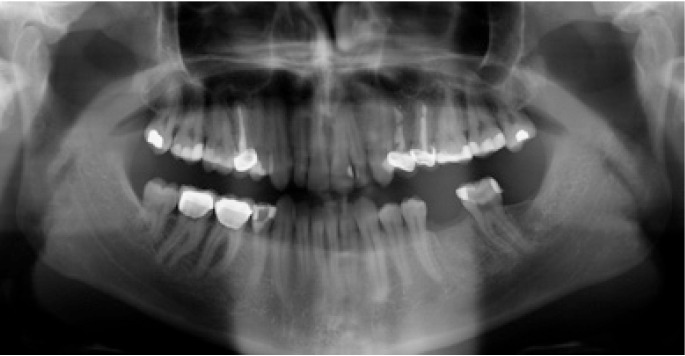
Preoperative panoramic X-ray view.

The following inclusion criteria were established: good general health, with no medical antecedents of relevance; single or partially edentulous segments not requiring simultaneous bone regeneration for the placement of implants (Figs. 1,2); and with a minimum follow-up of three months after implant placement. Patients with incomplete protocols or lacking the required follow-up were excluded. The following data were collected: patient age and gender, surgical technique, type of rehabilitation, splint adaptation and retention, fitting of the immediate aesthetics prosthesis, postoperative swelling and pain, peri-implant attached vestibular mucosal width, implant survival and follow-up.

-Surgical technique

**Figure 3:**
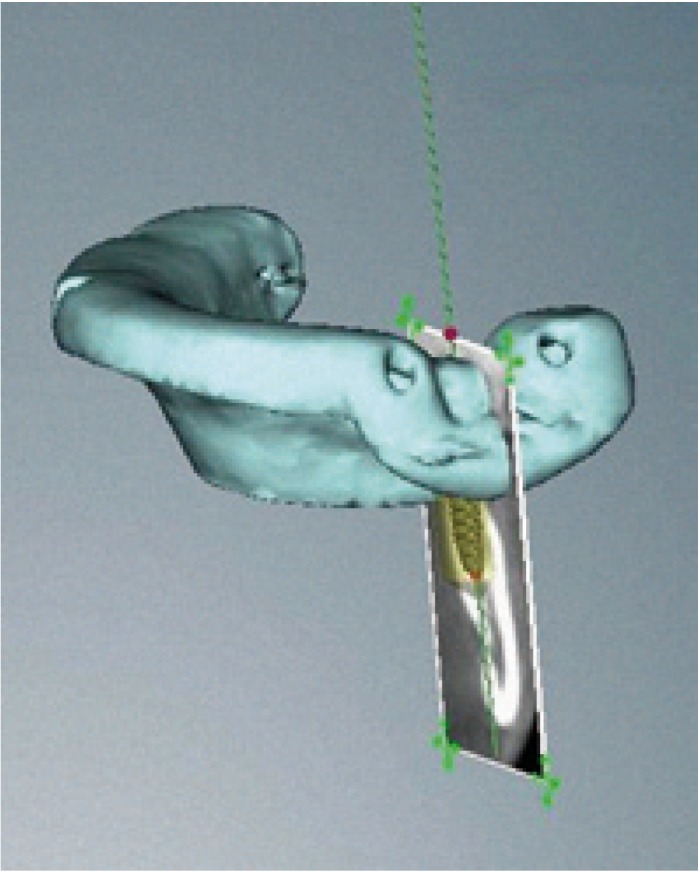
Planning with the NobelGuide® system. Note emergence of the implant in relation to the X-ray splint and the future prosthetic rehabilitation.

**Figure 4:**
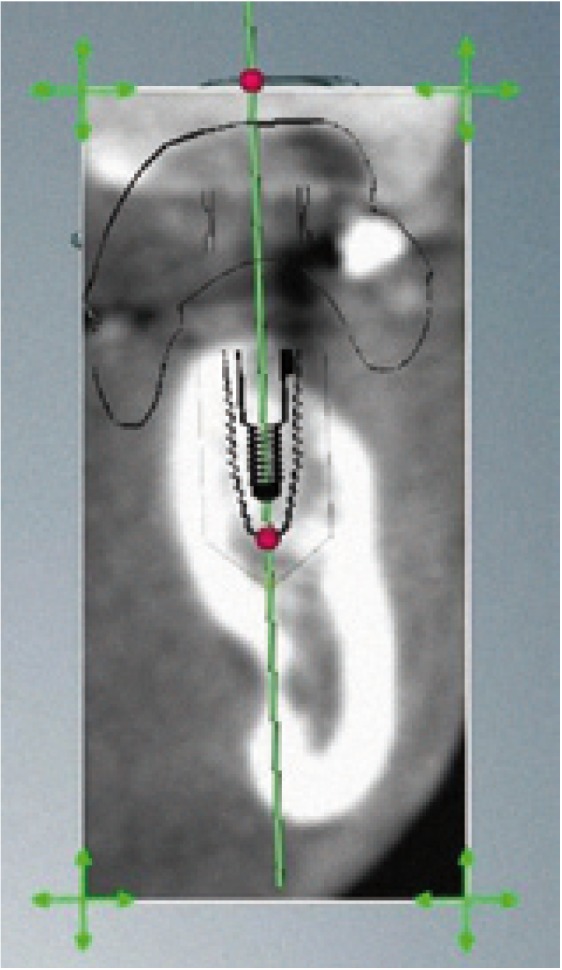
Implant location in the tomographic section.

**Figure 5:**
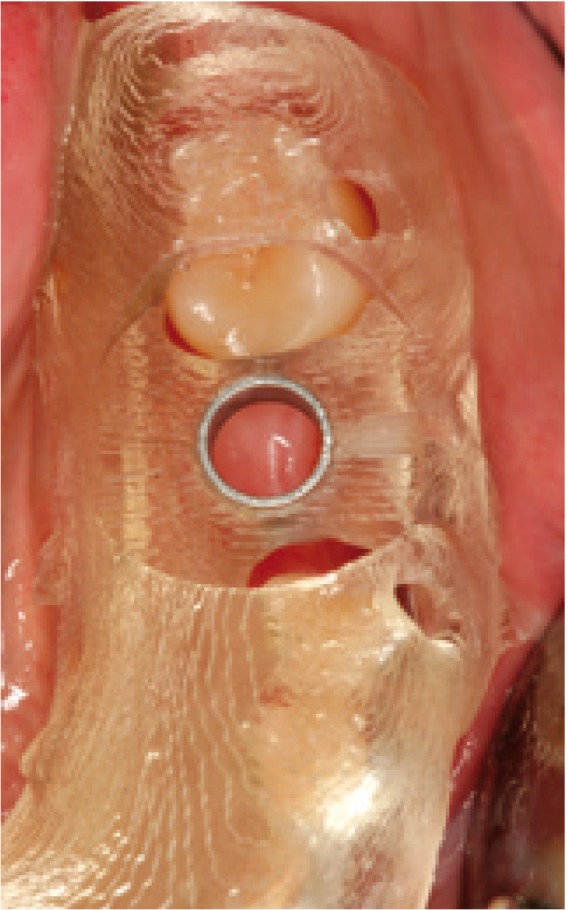
Intraoral view of the splint in the mouth.

**Figure 6:**
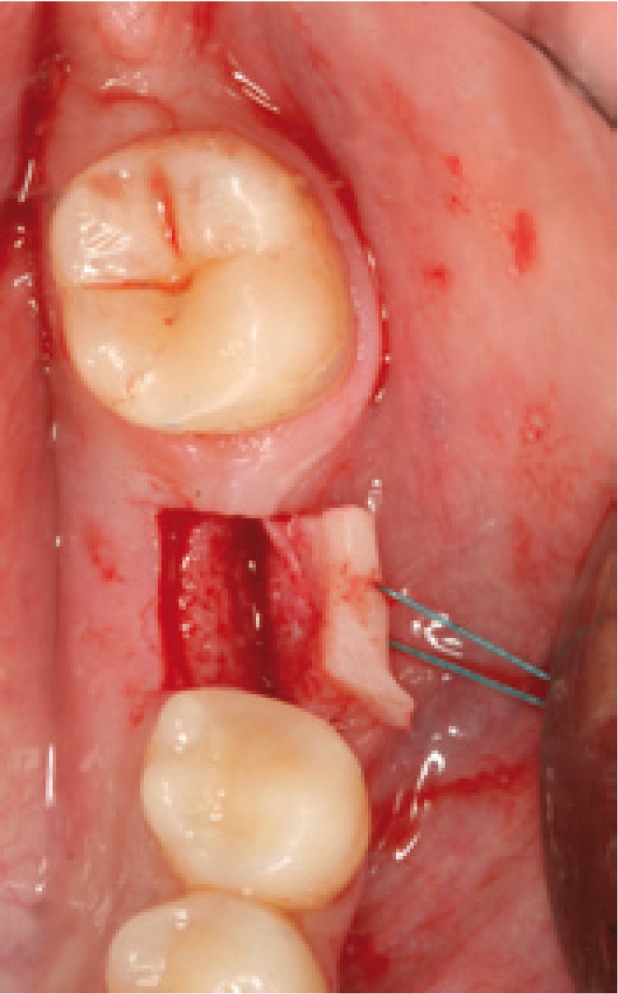
Raised mucoperiosteal flap. The flap limits comprise only emergence of the implant, with a one-millimeter safety peri-implant perimeter.

**Figure 7:**
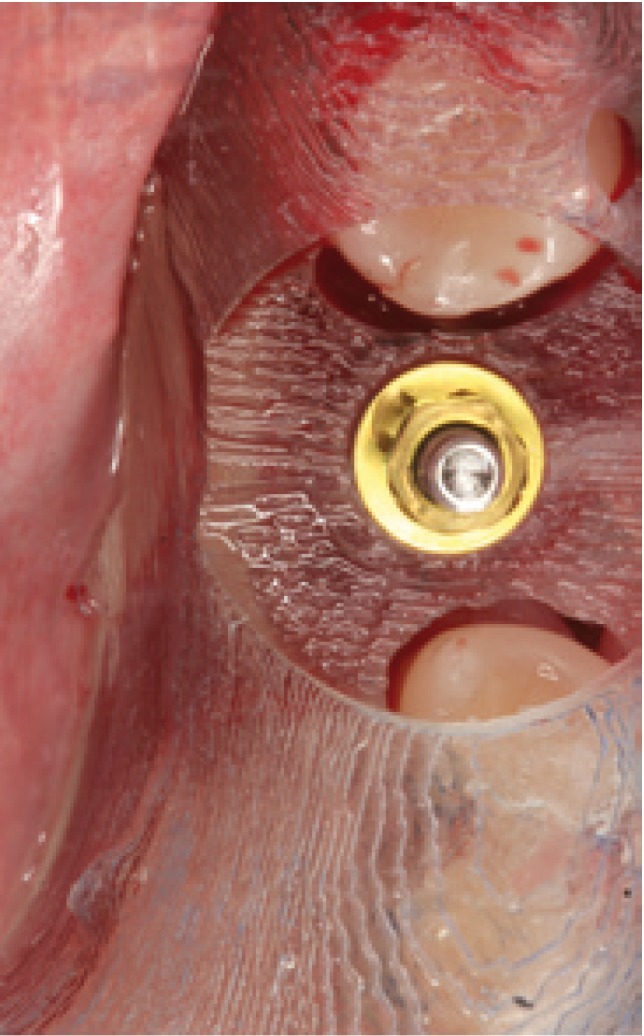
Implant placed with the help of the surgical guide.

**Figure 8:**
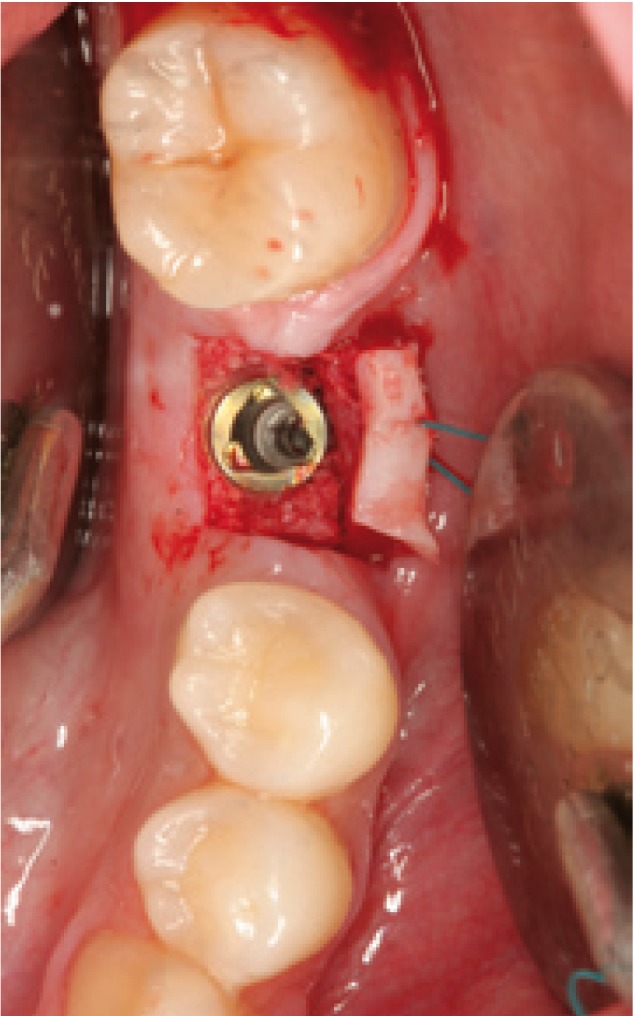
Following splint withdrawal the implant placed with guided surgery is observed, with preservation of the soft tissue – thus ensuring the presence of attached mucosa over the entire perimeter of the implant. Note that neither incision nor detachment include interproximal gingiva from the adjacent teeth.

**Figure 9:**
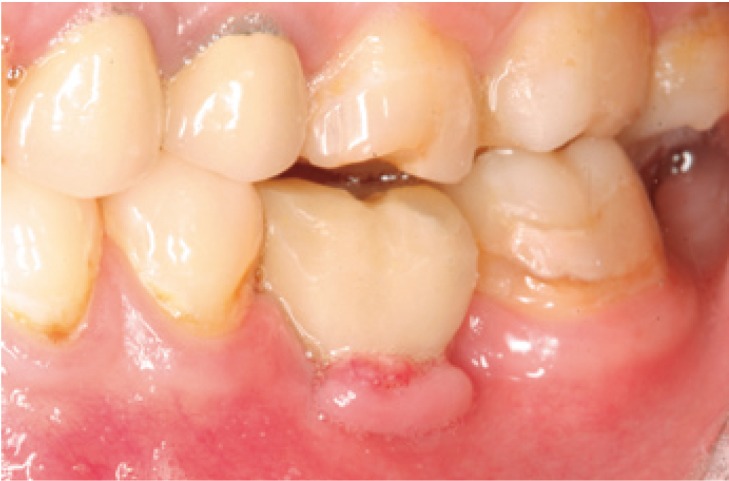
Unit screwed resin crown loaded on the implant. Note the attached vestibular mucosa of the implant.

**Figure 10:**
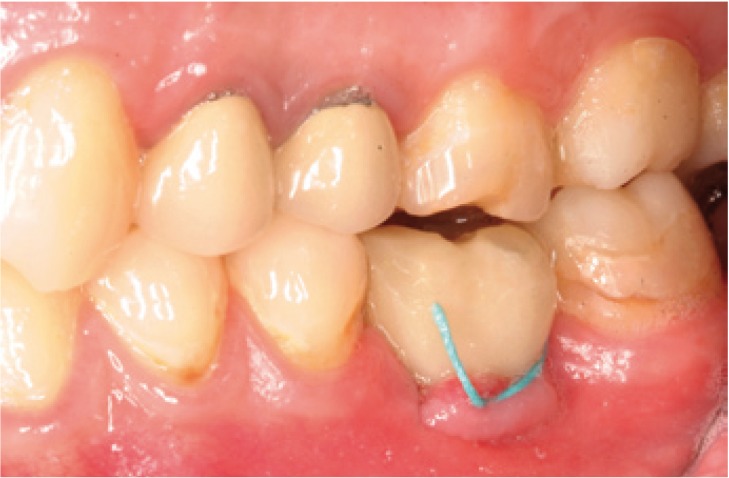
Miniflap suture.

**Figure 11:**
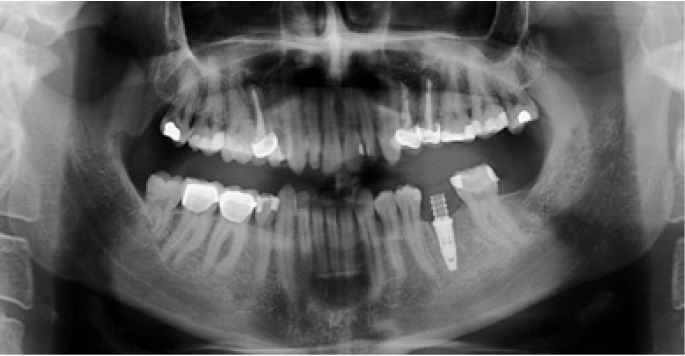
Postoperative panoramic X-ray view.

Surgery was carried out using the NobelGuide® guided surgery system (Nobel Biocare®, Göteborg, Sweden) in the study group, and the conventional technique in the controls. All patients in the study group underwent a cone beam computed tomography (CBCT) scan (Dental Picasso Master 3D, Ewoo Technology, Republic of Korea) with an X-ray splint. Posteriorly, implant surgery was planned (Figs. 3,4), and the manufacturer prepared a surgical splint. Fitting and retention of the latter was assessed. Surgery involved a modification of the Nobel Biocare® guided surgery technique, with the raising of a semicircular full-thickness miniflap. The procedure was as follows: a drill was used to eliminate the vestibular aspect of the surgical splint corresponding to the emergence of the implant, in order to raise the flap. With the splint in the mouth, the delimitation of the future incision was marked, ensuring that it precisely corresponded to the diameter of the implant. Following removal of the splint, the incision was made and the flap was raised vestibular (Figs. 5,6). The splint was posteriorly placed in the mouth and the usual guided surgery protocol was followed, though avoiding the punch drill (Fig. 7). After implant placement we removed the splint (Fig. 8), and a suture stitch was applied where necessary (Figs. 9,10,11).

-Prosthetic rehabilitation

Immediate aesthetics were established in the study group with implants placed at a torque of over 35 N/cm and without pathological occlusion. The provisional screwed resin prostheses were prepared before implant surgery, based on the surgical splints supplied by the manufacturer (Figs. 9,10). Fitting of the immediate prostheses was assessed. Imprints were obtained for the definitive cemented prostheses 8 weeks after implant placement, with fitting in the mouth after 10 weeks.

-Postoperative swelling and pain

The patients in both groups scored postoperative swelling and pain after 2, 6, 12 and 24 hours, and then once a day from day 2 to day 7 after surgery, based on a visual analog scale (VAS) from 0 (none) to 10 (very intense).

-Attached peri-implant mucosal width

Attached vestibular peri-implant mucosa width was evaluated in both groups 12 weeks after implant placement (2 weeks after fitting of the definitive prosthesis). A periodontal probe was used to measure from the gingival margin to the mucogingival line at the midpoint of the implant on the vestibular side.

-Follow-up

All patients in both groups were evaluated after one week, with recording of the VAS score and suture removal; after 10 weeks upon fitting the definitive prosthesis; and again after 12 weeks on measuring attached mucosa width.

## Results

Nineteen implants were placed in the study group: 14 as single rehabilitations and 5 as two partial restorations. Twenty-two implants were placed in the control group: 15 as single rehabilitations and 7 as three partial restorations. One implant failed in the study group (survival rate 94.8%), and another in the control group (survival rate 94.5%).

All surgical splints were fitted in the mouth: two without prior adjustments, and 10 with adjustments to ensure adequate fit. One splint was retained by itself, while 11 had to be grasped by an assistant. All implants in the study group were placed at a torque of 35 N/cm or more, with immediate aesthetics in each case. Sixteen immediate prosthetic rehabilitations were carried out: of these, 15 fitted adequately (with interproximal adjustments in 14 cases), while one did not.

The maximum pain intensity was recorded after 6 hours in both groups. The mean VAS score after 6 hours was 4 in the study group; 9 patients experienced pain while three did not. The mean VAS score after 6 hours was 4.9 in the control group; 11 patients experienced pain while one did not.

Maximum swelling in turn was recorded after 24 hours in the study group (mean VAS score 2.5); 9 patients suffered swelling while three did not. Maximum swelling was recorded on the second day in the control group (mean VAS score 3.4); 9 patients suffered swelling at that time, while three did not ( Table 1).

**Table 1:**
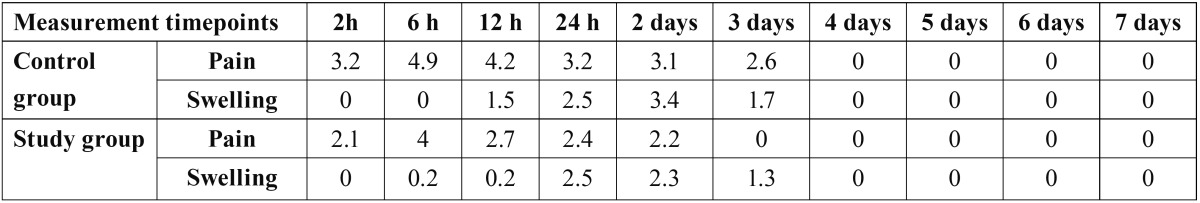
Mean pain and swelling scores in the control group and study group at the different timepoints evaluated.

The mean attached vestibular peri-implant mucosal width was 2.9 mm in the study group (range 1-4 mm) versus 3.2 mm in the control group (range 2-5 mm) three months after implant placement (2 weeks after placement of the definitive prosthesis).

## Discussion

A systematic review published in 2009 reported a 96.7% survival rate among 506 implants placed with guided surgery (12). In the present study the survival rate among 19 implants was 94.8% after three months. Of the 16 immediate rehabilitations performed, 15 fitted adequately, with the need for adjustments in 14 cases. This complication of guided surgery has been well documented in the literature (13,14).

Flapless surgery offers a number of advantages, including a better postoperative course for the patient, and a shortening of the surgical times. These aspects have been documented by a series of metaanalyses (15). Since flapless implant placement consti-tutes blind surgery, it may prove complicated in the absence of a guiding system (16). A number of studies comparing postoperative swelling and pain in flapless guided and normal surgery have reported an improved postoperative course when flapless guided surgery is used for implant placement (17). A recent study has compared pain, analgesic consumption and surgical time in conventional implant placement versus guided surgery with and without the raising of a flap. All the studied parameters yielded better results with flapless guided surgery, though no advantages were noted for guided surgery with the raising of a flap and conventional implant placement (18). In our study the postoperative course proved slightly better in the patients subjected to guided surgery than in those subjected to conventional implant placement with the raising of a flap.

A circular scalpel is usually employed in flapless guided surgery; case selection therefore should be very strict from the mucogingival point of view if the implant is to be surrounded by attached gingiva (6). In our modification of the technique we raise a miniflap that only very slightly exceeds the flap raised with the circular scalpel, without implicating the periodontal soft tissues of the adjacent teeth; in this way mucosal attachment is maintained, and we can avoid the negative postoperative effects associated with the raising of large flaps. From the mucogingival perspective, the results obtained are very similar in both the study group and in the control group – with attached vestibular peri-implant mucosa in both groups and a slightly greater mucosal width in the control group.

In this preliminary study, guided implant surgery with a semicircular miniflap in single and partial replacements resulted in slightly less postoperative pain and swelling than with the conventional implant technique. The attached vestibular mucosa width was greater in the control group, though the differences were very small.
